# Seismic Behavior of Steel Column Base with Slip-Friction Connections

**DOI:** 10.3390/ma13183986

**Published:** 2020-09-09

**Authors:** Chengyu Li, Qi Liu, Gongwen Li

**Affiliations:** School of Urban Construction, Wuhan University of Science and Technology, Wuhan 430065, China; lichengyu@wust.edu.cn (C.L.); lq12345890@163.com (Q.L.)

**Keywords:** column base, slip-friction connection, hysteretic behavior, damage-free energy dissipation

## Abstract

Traditional rigid column base connections are damaged to different degrees after an earthquake and the damage is generally nonrecoverable. Thus, the cost of repairing or dismantling is quite high. A new type of slip-friction column base connection is proposed in this paper, which aims to replace the yielding energy dissipation of the traditional column base connection by the sliding friction energy dissipation between the arc endplates, thus achieving the design objective of damage-free energy dissipation. Finite element simulation was conducted to study the hysteretic performance of the proposed connections considering different axial compression ratios. The research indicates that both kinds of the proposed connections show good energy dissipation behavior and the increase of axial compression force can increase the energy dissipation ability. It also shows that the two kinds of connections can achieve the objective of damage-free energy dissipation. For the proposed connection, future research is still needed such as corresponding tests in the laboratory, the effect of the connection on the whole structure, and the re-centering systems for the connections.

## 1. Introduction and Background

Steel structure has been widely used in high-frequency seismic regions because of its excellent performances in previous earthquakes. However, in the 1994 Northridge Earthquake [1] in the United States and the 1995 Hanshin Earthquake [2] in Japan, traditional rigid column base connections have been damaged to varying degrees, and the cost of repairing or dismantling after an earthquake is very high. Yang et al. [3], Nakashima et al. [4], and Borzouie [5] conducted a series of cyclic performance tests of traditional exposed column base connections. The test results show that the yielding of column base connections will produce residual deformation at the top of the column, and the axial shortening due to the yielding of column base can cause undesirable effects in the building during earthquake excitation [6]. Moreover, it is difficult for the building structure to recover to the initial vertical state when yielding occurs. Therefore, it is very difficult to repair or remove the traditional column base connections after the first floor is damaged. Akira et al. [7] pointed out that whether the damaged parts are repaired or dismantled after an earthquake, it will cause great waste of resources and social labors.

**Figure 1:**
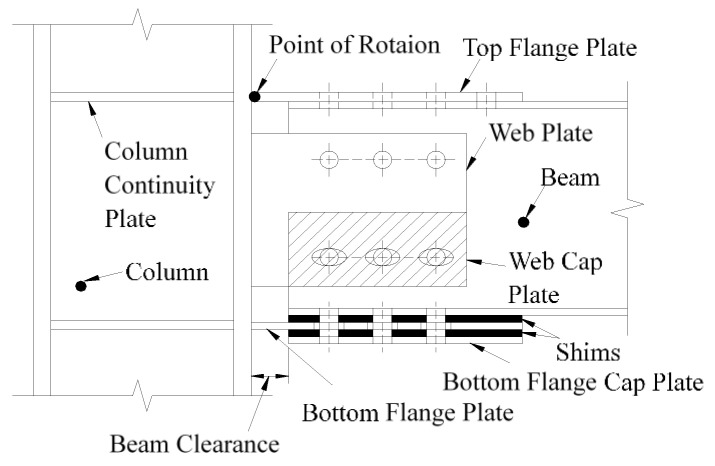
Beam-column asymmetric friction connection (AFC) [9].

**Figure 2:**
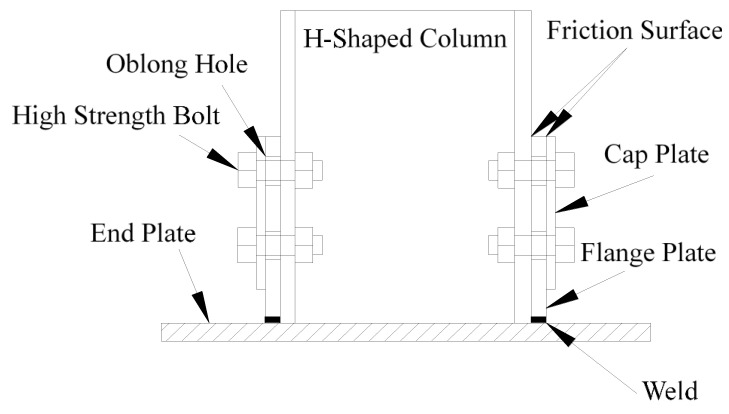
Column base friction connection (adapted from [17]).

Thus, to solve this problem, the concept of a low damage connection is proposed to dissipating seismic energy by setting auxiliary energy-consuming components to keep the main structure from damage. One of the most commonly used low damage connections is friction connection and it has been studied by many scholars. The sliding hinge joint (SHJ) beam-column friction connection which is known as asymmetric friction connection (AFC) was proposed by Clifton [8] for the first time (Figure 1 [9]) and it has been further developed by many scholars [9,10,11,12,13,14,15,16]. This kind of AFC beam-column connection generally uses friction damper to replace the traditional rigid structure connections. Through pushover analysis, this kind of connection can control the structural damage, while the residual lateral displacement still exists after a large earthquake. For column base friction connections (Figure 2 [17]), Borzouie et al. [17] conducted experimental studies on the cyclic performance of column base strong and weak axis aligned asymmetric friction connection, and it was found that the proposed details for base connections with friction connections provide repeatable cycles without strength degradation or any requirements for repair or replacement following an earthquake. Freddi et al. [18] and Dimopoulos et al. [19] also proposed a new type of a sway damage-free steel column base connection and the results of finite element simulation verified the accuracy of the *M*-*θ* curve. This kind of connection can effectively avoid yielding of the column base, thus, achieving the objective of damage-free energy dissipation. Borzouie et al. [20] also conducted cyclic tests on Grade 10.9 bolts in AFC. The test results showed that Grade 10.9 bolts had less ductility compared to Grade 8.8 bolts, and Grade 10.9 bolts fractured for large sliding displacements. Thus, Grade 8.8 bolts are used in this paper.

**Figure 3:**
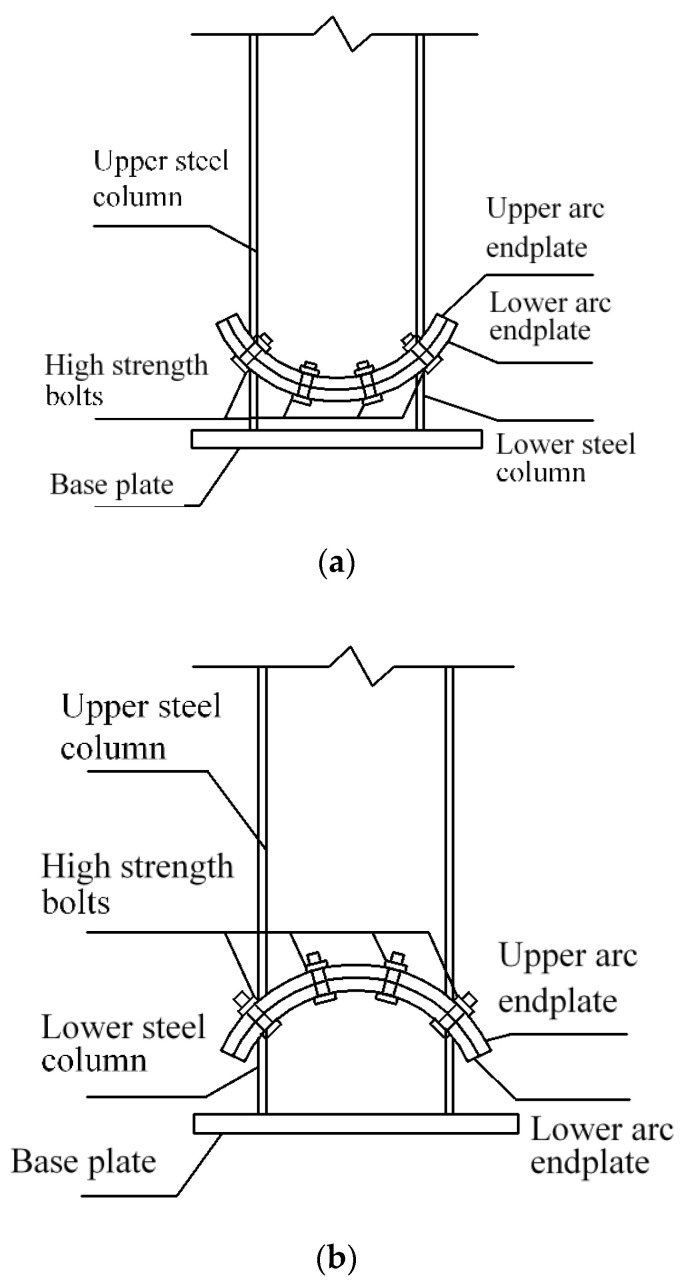
Steel column base with slip-friction arc endplates: (**a**) concave connection; (**b**) convex connection.

On the basis of previous studies, a new kind of steel column base with slip-friction arc endplates is proposed (as shown in Figure 3). For this kind of connection, if the moment under the horizontal load is less than the slip moment, the connection does not rotate and it behaves like the ordinary column base connection. However, when the moment exceeds the slip moment, the connection rotates. Under large earthquakes, the connection dissipates seismic energy through the rotational friction between the arc endplates and the friction between the arc endplates and bolts. The sliding friction energy dissipation is used to replace the energy dissipation caused by material yielding of the traditional connection. Compared to the AFC introduced above (Figure 1 and Figure 2), the axial compression force transferred from the column may increase the friction force between the arc endplates. Thus, the proposed connection may have better energy dissipation behavior.

As is shown in Figure 3, the connection is composed of steel column, upper and lower arc endplates, high-strength bolts, and base plate. Among them, the steel column and the arc endplates are welded together, the upper arc endplate and the lower arc endplate are connected by high-strength bolts, and the pre-pressure between the upper and lower arc endplates is produced by applying a pretension force to high-strength bolts. The upper arc endplate is provided with oblong bolt holes so that the connection can rotate along the oblong holes, and the lower arc endplate is provided with ordinary circular bolt holes. Under horizontal external force, when the moment reaches the slip moment, the upper steel column and the upper arc endplate will rotate, and the relative slip between the upper and lower arc endplates will cause friction. The maximum rotation angle of the upper steel column and the upper arc endplate can be controlled by the size of the oblong bolt hole opened on the upper arc endplate. The connection can be divided into a concave connection (Figure 3a) and a convex connection (Figure 3b) according to the orientation of arc endplates.

## 2. Objectives

The main purpose of this paper is to study the seismic behavior of a steel column with the proposed slip-friction connections to check the ability of damage-free energy dissipation. As is introduced above, finite element analysis (FEA) is a commonly used method to study the seismic behavior of steel column base connections. Thus, cyclic FEA simulation of the proposed connection will be conducted to study the energy dissipation and seismic behavior. Furthermore, the seismic behavior of concave and convex connections will be compared in this paper.

## 3. Finite Element Analysis 

### 3.1. Size of Specimen

**Figure 4:**
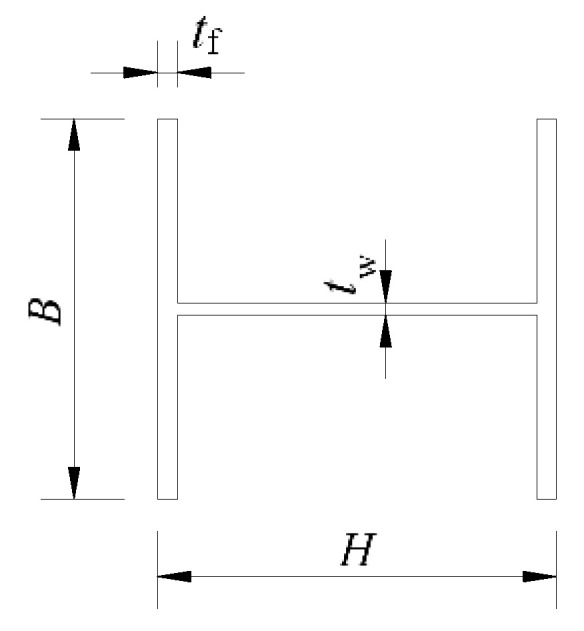
Size of H-shaped section.

**Figure 5:**
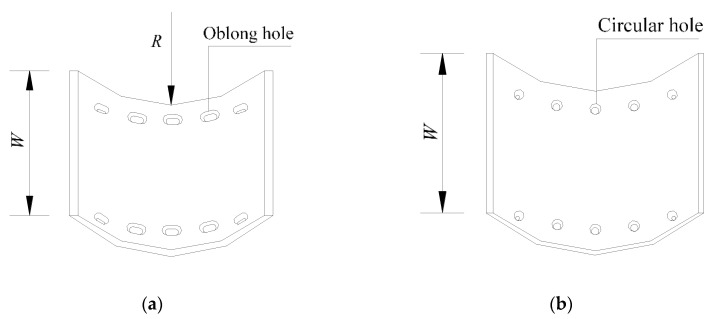
Holes in arc endplates: (**a**) upper arc endplate; (**b**) lower arc endplate.

**Figure 6:**
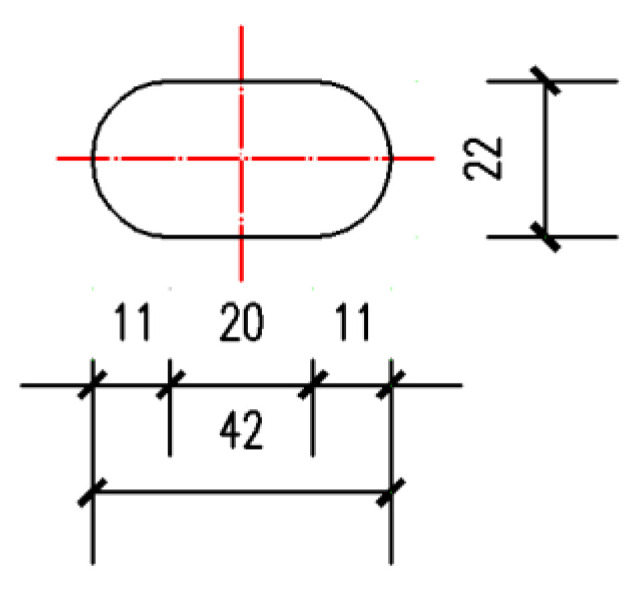
Size of oblong bolt holes (unit: mm).

The section of the specimen used in FEA is H 300 mm × 300 mm × 10 mm × 15 mm, which means the section is H-shaped (Figure 4) and the height (*H*) is 300 mm, the width of the flange (*B*) is 300 mm, the thickness of the web (*t*_w_) is 10 mm, and the thickness of the flange (*t*_f_) is 15 mm. The total length of the specimen is 1700 mm. As is shown in Figure 5, the inner radius (*R*) of the upper arc endplates is 0.7 *H*, the width of the arc endplates (*W*) is 500 mm, the thickness of the arc endplates is 20 mm, the length of oblong bolt holes (Figure 5a) in the upper arc endplate is 42 mm (Figure 6), and the diameter of circular bolt holes (Figure 5b) in the lower arc endplate is 22 mm.

### 3.2. Material Model

**Figure 7:**
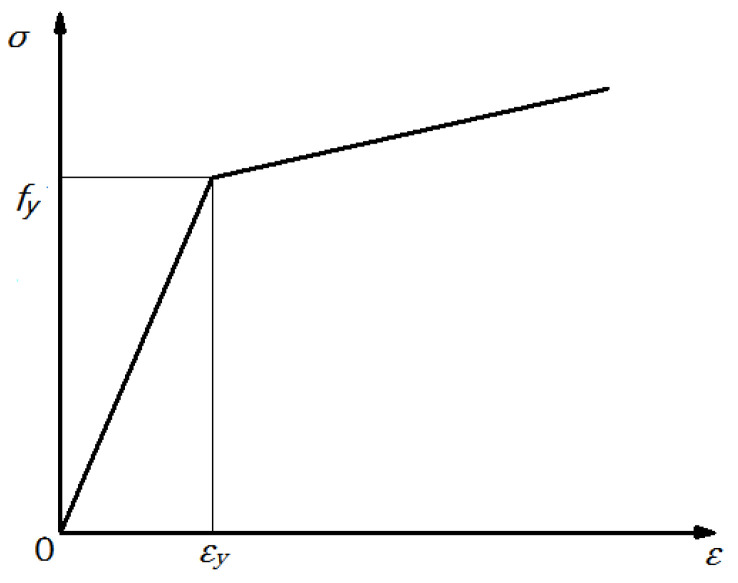
Stress–strain relationship of the steel material.

The steel column and arc endplates are made of Q345B steel, which means the yield strength of the steel is 345 Mpa. The bolts are 8.8 grade M20 high-strength bolts, which means the tensile strength is 800 MPa, the yield strength ratio is 0.8, and the nominal diameter of the bolt is 20 mm. Finite element analysis involves the plastic development of materials and the influence of material nonlinearity and geometric nonlinearity. The corresponding parameter settings in the ANSYS (Version 12.0, Swanson Analysis Systems Inc., Houston, TX, USA) [21] analysis are shown in Table 1. The stress–strain relationship of the steel material is shown in Figure 7. The von Mises yield criterion is selected, the Poisson’s ratio is set as 0.3, and the strengthening model MKIN (multilinear kinematic hardening) is adopted in the analysis model.

### 3.3. Finite Element Model

**Figure 8:**
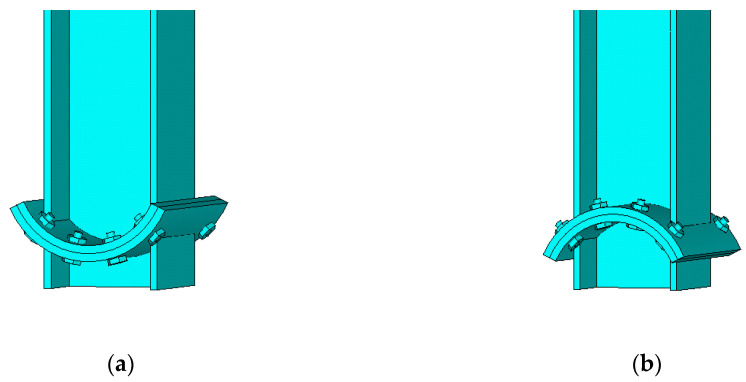
The finite element analysis (FEA) model of connections: (**a**) concave connection; (**b**) convex connection.

**Figure 9:**
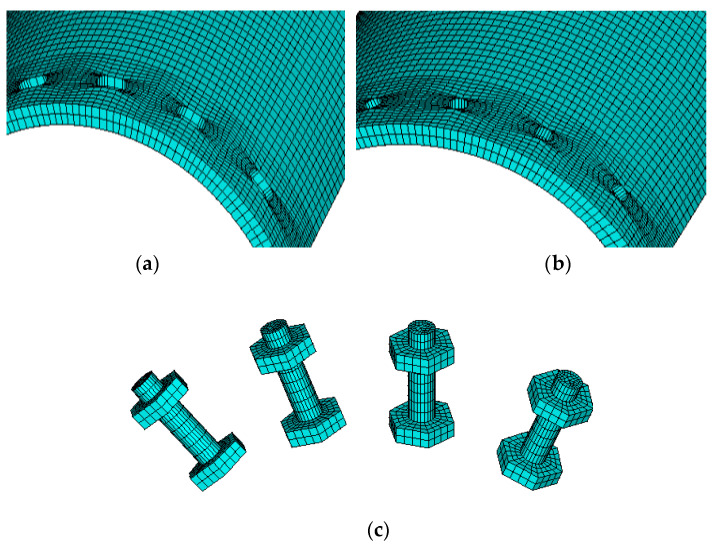
Element meshing of arc endplates and bolts: (**a**) upper arc endplate; (**b**) lower arc endplate; (**c**) high-strength bolts.

The FEA model before meshing is shown in Figure 8. All the components here, such as high-strength bolts and arc endplates, are built by solid elements Solid185. The contact elements CONTA174 and TARGE170 are used to simulate the contact between the two arc endplates and between arc endplates and bolts. The pre-tension element PRETS179 is used on the top area of the high-strength bolts to apply the pre-tension force. The element meshing of the arc endplates and bolts is shown in Figure 9.

### 3.4. Constraints and Loading

The friction model used here is the “extended Lagrange” friction formula, and the friction coefficient is set as 0.4. Binding constraints are used to simulate the welding effect, such as upper steel column and upper arc endplate, lower steel column and lower arc endplate, as well as screw and nut.

The fixed constraint UX = UY = UZ = RX = RY = RZ = 0 is applied to the bottom of the base plate. In order to apply the vertical load and horizontal load on the top of the upper steel column, a rigid cover plate is set as the loading plate on the top of the column in the model. The length and width of the loading plate are the same as the upper steel column, and the thickness is 10 mm. The material of the cover plate is set as ideal linear elastic, and the elastic modulus is 10^3^ times the elastic modulus of the steel column.

**Figure 10:**
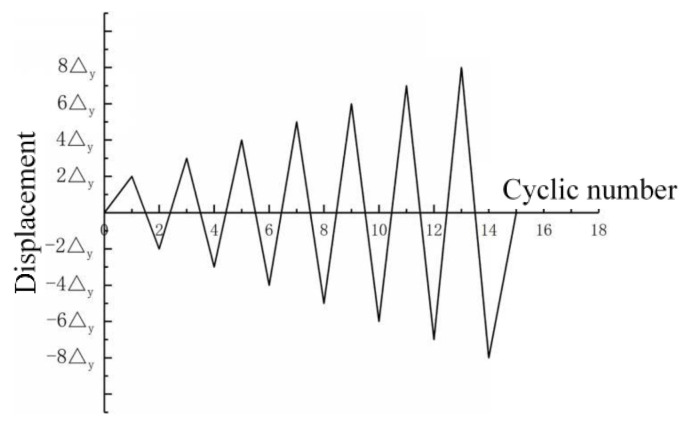
Loading history of horizontal displacement.

After the modeling of the specimen, the loads are applied in steps. Firstly, the pretension force of 125 kN is applied to the high-strength bolts. Then, an axial vertical load (shown in Table 2) is applied to the top of the upper steel column, and the applied axial load is converted into a surface load, which is applied to the loading plate on the top of the column. In Table 2, *n* is the axial compression ratio. The cyclic horizontal displacement is applied to the top of the column and the loading history is shown in Figure 10, where Δ_y_ is the displacement of the top of the column corresponding to the rotation angle of 0.006 radian.

## 4. Results and Discussion

### 4.1. Rotation Center

**Figure 11:**
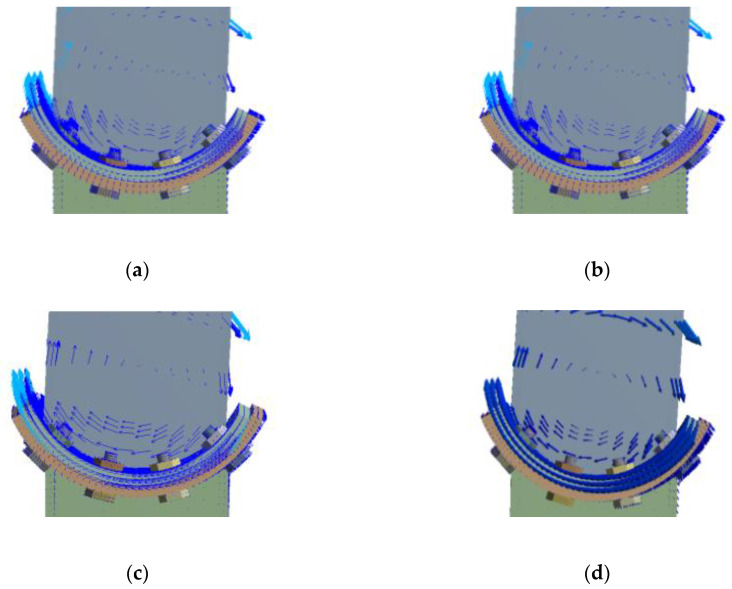
Rotation vector diagram of concave connections: (**a**) *n* = 0; (**b**) *n* = 0.1; (**c**) *n* = 0.2; (**d**) *n* = 0.3.

The rotation vector diagram of the concave connection is shown in Figure 11. The ideal rotation center is the center of the arc endplate, while it can be observed that during the rotation of the connection, the position of the rotation center does not coincide with the ideal rotation center and it drifts with the rotation of the steel column. It can also be observed that the rotation center moves closer and closer to the ideal rotation center when the vertical axial load is increased.

**Figure 12:**
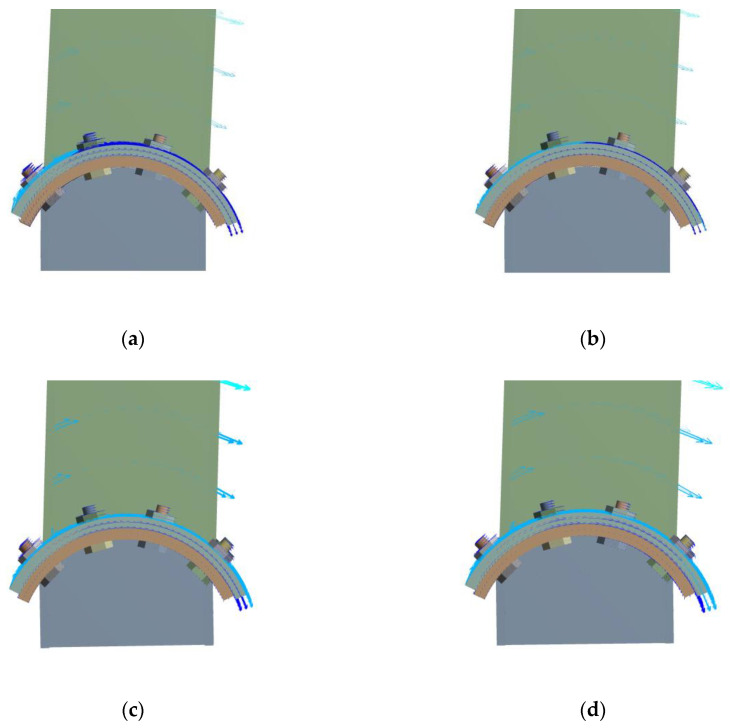
Rotation vector diagram of convex connections: (**a**) *n* = 0; (**b**) *n* = 0.1; (**c**) *n* = 0.2; (**d**) *n* = 0.3.

Figure 12 shows the rotation vector diagram of the convex connection. It can be observed that there is no drifting of the rotation center during the rotation of the convex connection, and the position of the rotation center is consistent with the ideal rotation center for all the specimens, which means that the vertical load has no effect on the position of the rotation center.

### 4.2. M-θ Curve and Energy Dissipation Behavior

**Figure 13:**
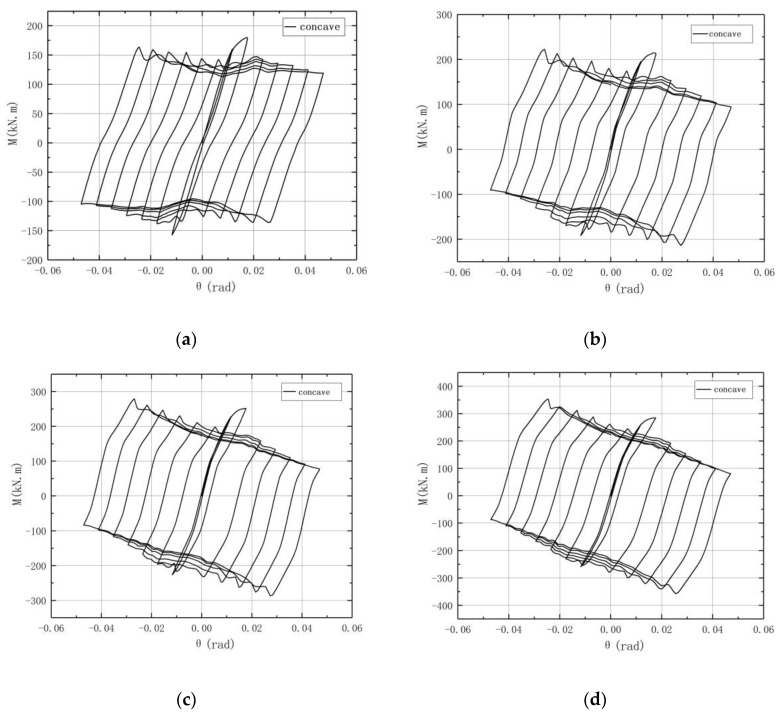
*M*-*θ* curves of concave connections: (**a**) *n* = 0; (**b**) *n* = 0.1; (**c**) *n* = 0.2; (**d**) *n* = 0.3.

The *M-θ* curve for the concave connection is shown in Figure 13, where *M* is the moment at the rotation center and *θ* is the rotation angle of the connection. It can be observed that during the initial stage of the loading, the *M-θ* curve basically linearly increases, and the connection is in elasticity, while when the slip moment is reached, the connection starts to rotate, and a slipping segment appears. It is shown in Figure 13 that the *M-θ* curve for each circle is a parallelogram, which means that it has a good energy dissipation behavior. It can also be observed that after the second circle, the peak load corresponding to the positive radian of each circle decreases step by step, and the degree decreases as the vertical load increases. Moreover, as the vertical load increases, the peak load of the second circle increases gradually and the wave-like jitter decreases.

**Figure 14:**
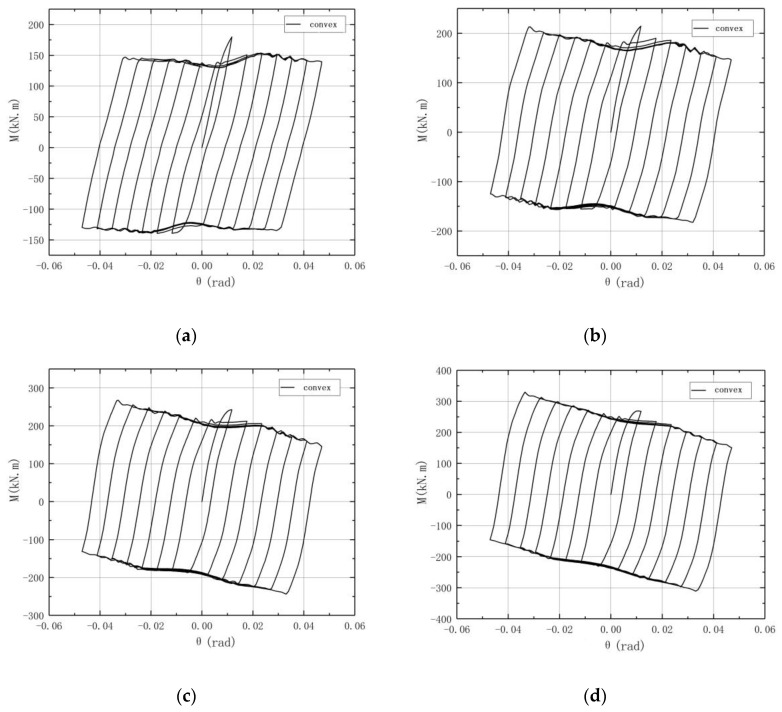
*M*-*θ* curves of convex connections: (**a**) *n* = 0; (**b**) *n* = 0.1; (**c**) *n* = 0.2; (**d**) *n* = 0.3.

The *M-θ* curve of the convex connection is shown in Figure 14. It can also be observed that the shape of the hysteresis loop is close to a parallelogram, which reflects the large slip deformation characteristics of the convex connection. As the vertical load increases, the curve shows a downward trend similar to the concave connection, while the curve of the convex connection is smoother and the curves after the second circle nearly overlap, especially for the specimen under a high vertical load. It can be seen from the *M-θ* curves that the hysteresis loop under the cyclic load is plump, and the large hysteresis loop area indicates that the convex connection has good energy dissipation ability.

The hysteresis loop of the convex connection also shows a “small wave” jittering, which may be related to the slight deformation of the upper arc endplate. As the vertical load increases, the *M-θ* curves become smoother.

**Figure 15:**
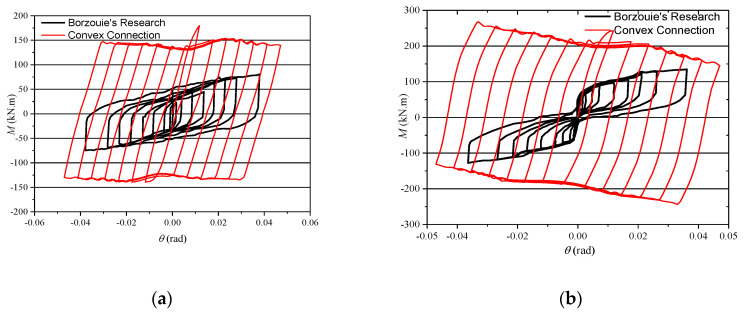
*M*-*θ* curves of base bending about the strong axis: (**a**) *n* = 0; (**b**) *n* = 0.2 (adapted from [17]).

Figure 15 shows the outline of typical *M*-*θ* curves of column base AFC in Borzouie’s research [17]. Comparing the curves in Figure 15 with those in Figure 13 and Figure 14, it can be concluded that the hysteresis loops of the proposed connections are plumper, especially when *n* = 0.2, which means a better energy dissipation ability. This is because the axial compression force transferred from the column may increase the friction force between the arc endplates for the proposed connections.

It can also be observed in Figure 13 and Figure 14 that when *θ* reaches 1/50, which is the limited inter-story displacement angle under large earthquakes in the Chinese code [22], the bearing capacity and energy dissipation ability of the column remains steady, which means that the proposed connections have good seismic behavior under large earthquakes, and the limitation of a displacement of structure with the proposed connections could be designed according to the existing code [22].

**Figure 16:**
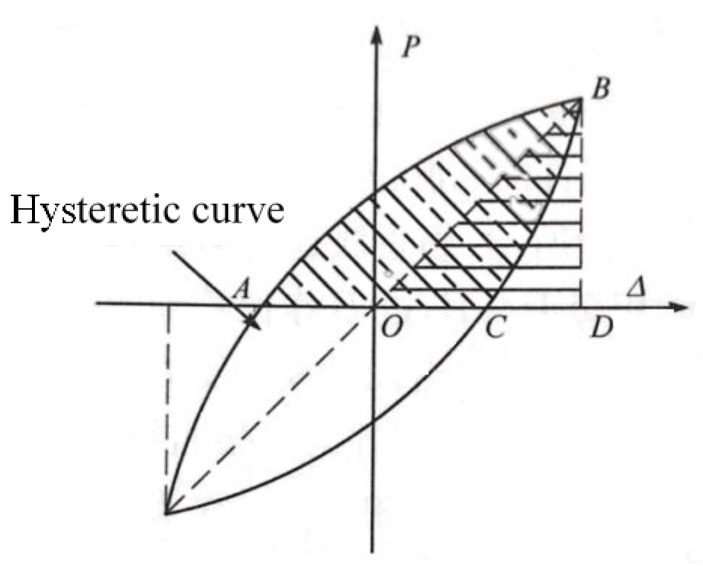
Definition of equivalent viscous damping ratio.

To study the effect of vertical load on the energy dissipation ability of the connections, the equivalent viscous damping ratio is calculated for convex connections. The equivalent viscous damping ratio *h*_e_ is calculated by the following formula:

(1) $$h_{e}=\frac{1}{2\pi }\frac{S_{ABCOA}}{S_{\Delta BOD}},$$

where *S_ABCOA_* and *S_BOD_* are shown in Figure 16. The calculated *h*_e_ is listed in Table 3. It is shown in Table 3 that *h_e_* increases as the axial compression ratio increases, which indicates that the increase of axial force may increase the energy dissipation ability of the connections.

### 4.3. Deformation of the Arc Endplates

**Figure 17:**
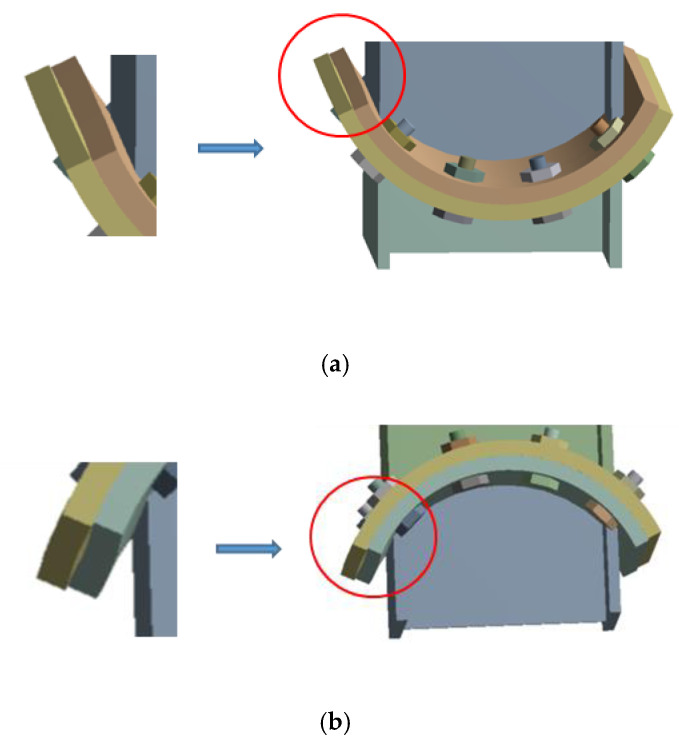
Deformation of the arc endplates: (**a**) concave connections; (**b**) convex connections.

It can be observed in the FEA that there is a small opening between the upper arc endplate and lower arc endplate (Figure 17). It can be observed from Figure 17 that the deformation at the center along the width direction of the arc plate is the largest. The maximum deformation Δ_s1max_ of the upper arc plate under a different axial compression ratio is listed in Table 4. It is shown in Table 4 that Δ_s1max_ decreases as the axial compression ratio increases and the maximum value of Δ_s1max_/*W* is 1/647. It can be observed from the FEA that the arc endplates are under an elastic state during the whole loading process.

### 4.4. Working State of Bolts

**Figure 18:**
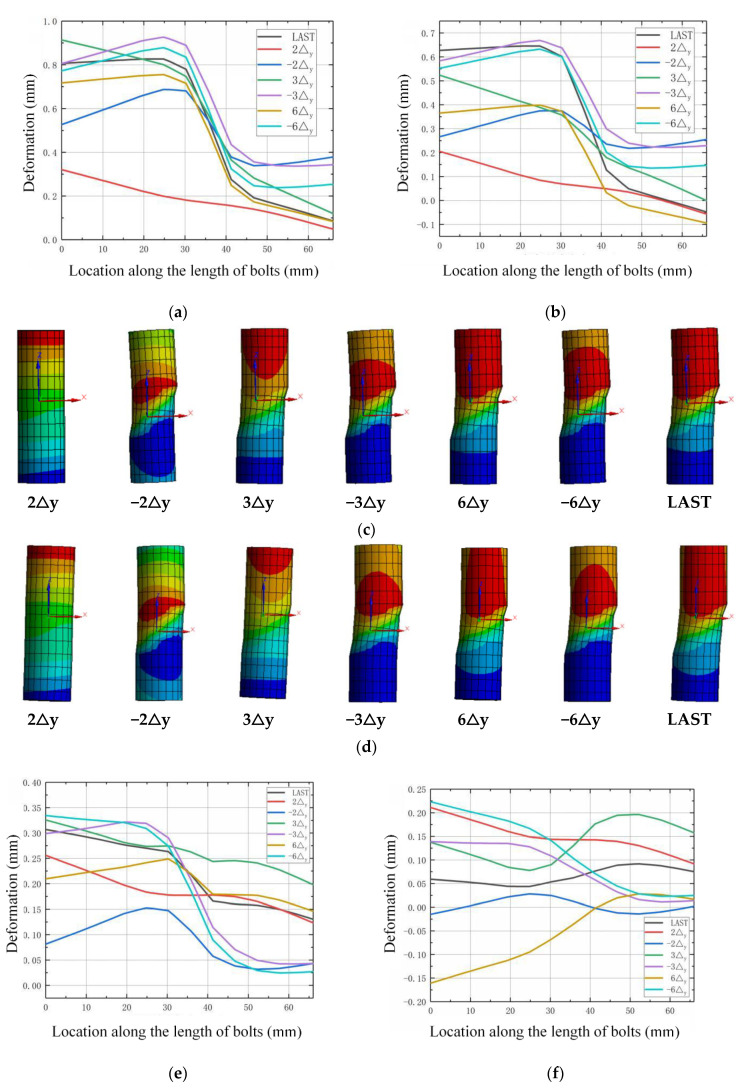

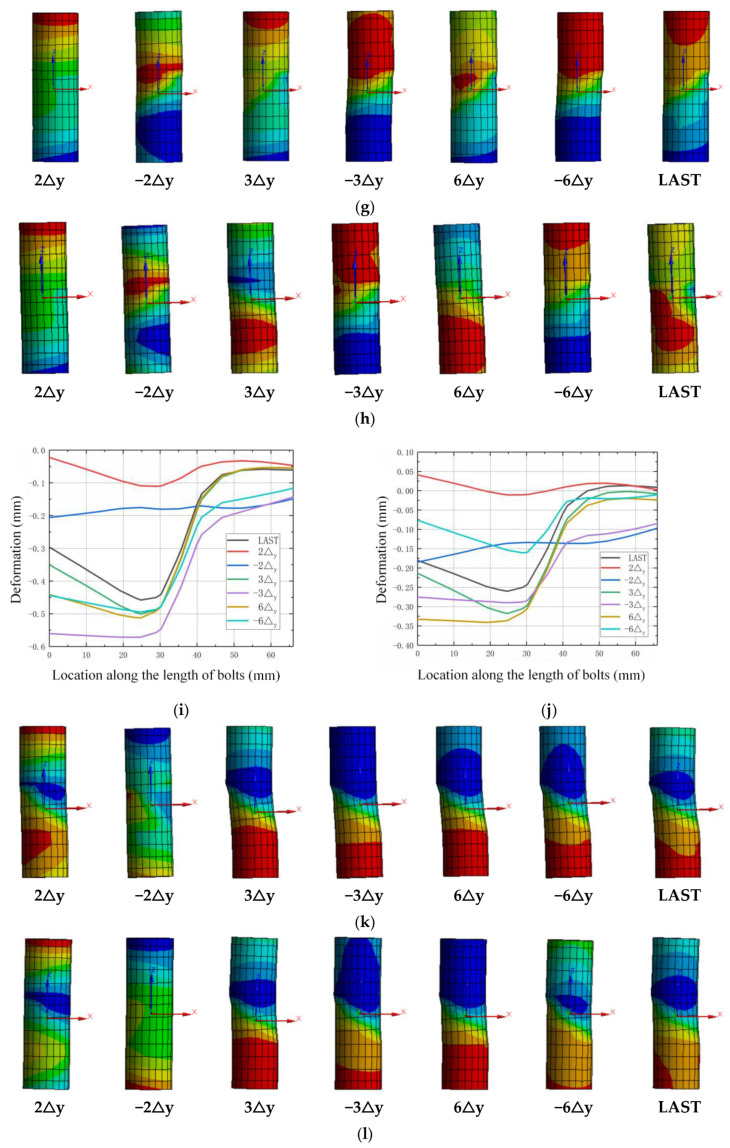

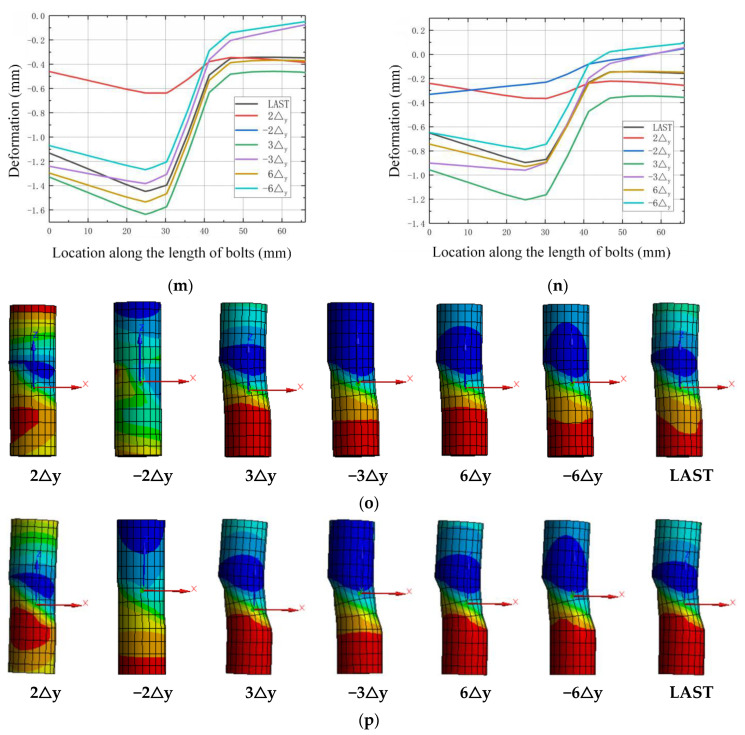
Deformation of the bolts for concave connections: (**a**) *n* = 0, bolts 1; (**b**) *n* = 0.2, bolts 1; (**c**) *n* = 0, development of deformation for bolts 1; (**d**) *n* = 0.2, development of deformation for bolts 1; (**e**) *n* = 0, bolts 2; (**f**) *n* = 0.2, bolts 2; (**g**) *n* = 0, development of deformation for bolts 2; (**h**) *n* = 0.2, development of deformation for bolts 2; (**i**) *n* = 0, bolts 3; (**j**) *n* = 0.2, bolts 3; (**k**) *n* = 0, development of deformation for bolts 3; (**l**) *n* = 0.2, development of deformation for bolts 3; (**m**) *n* = 0, bolts 4; (**n**) *n* = 0.2, bolts 4; (**o**) *n* = 0, development of deformation for bolts 4; (**p**) *n* = 0.2, development of deformation for bolts 4.

**Figure 19:**
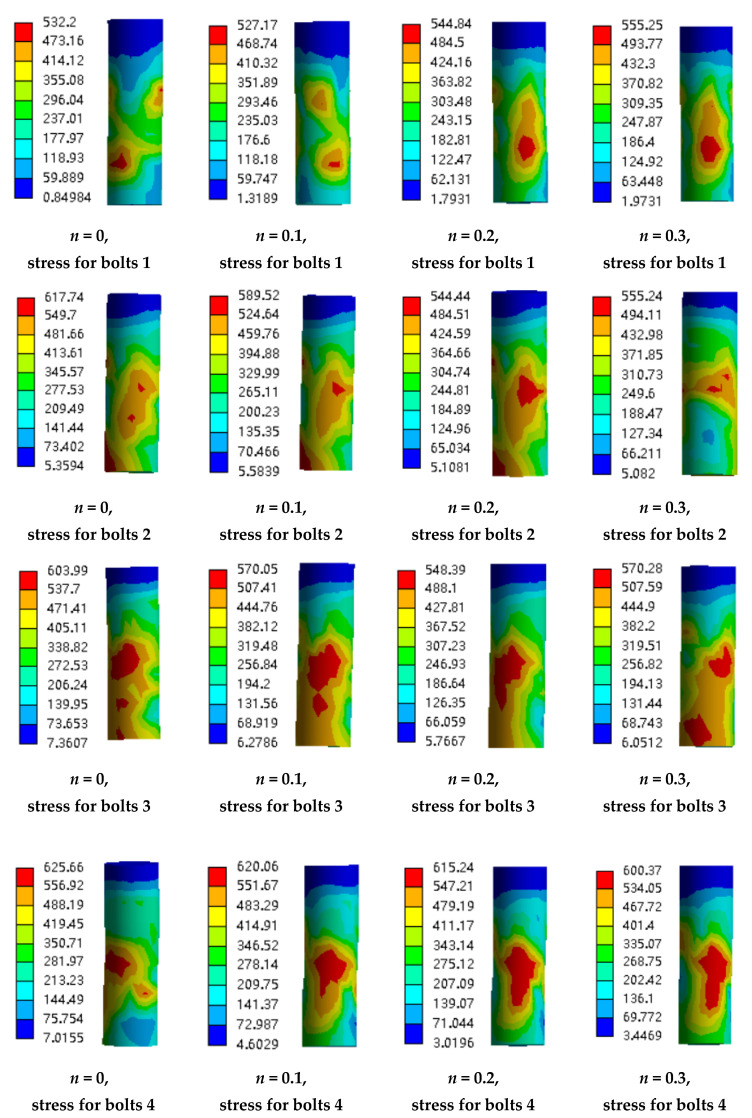
The von Mises stress of the bolts for concave connections.

**Figure 20:**
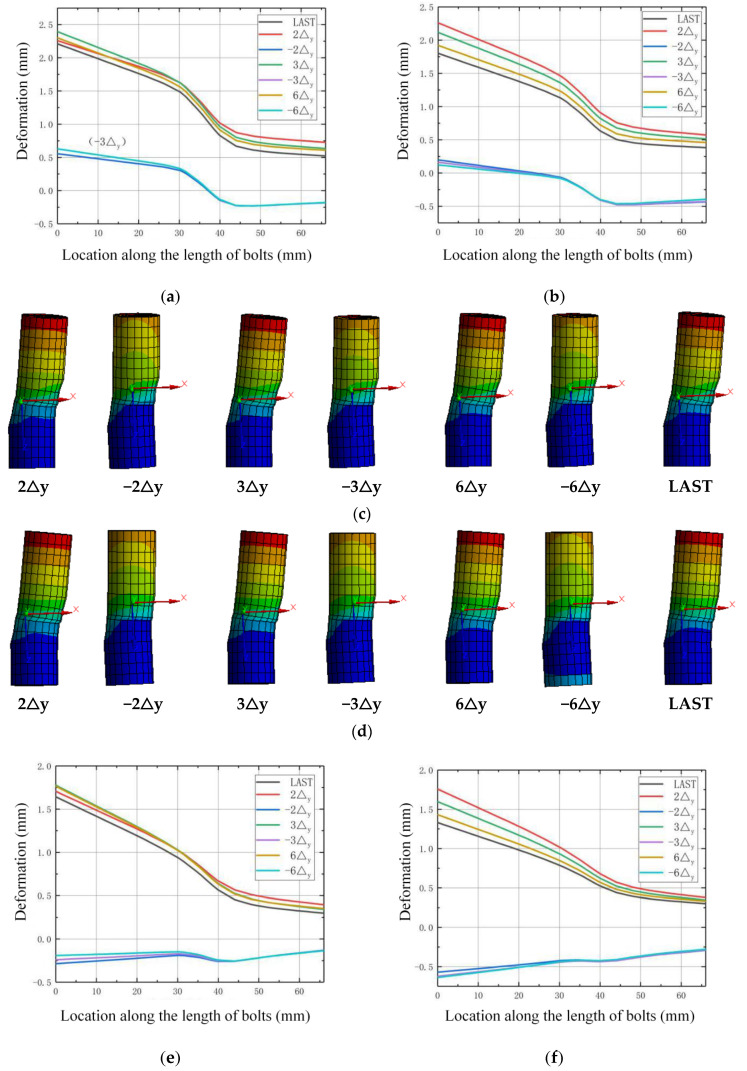

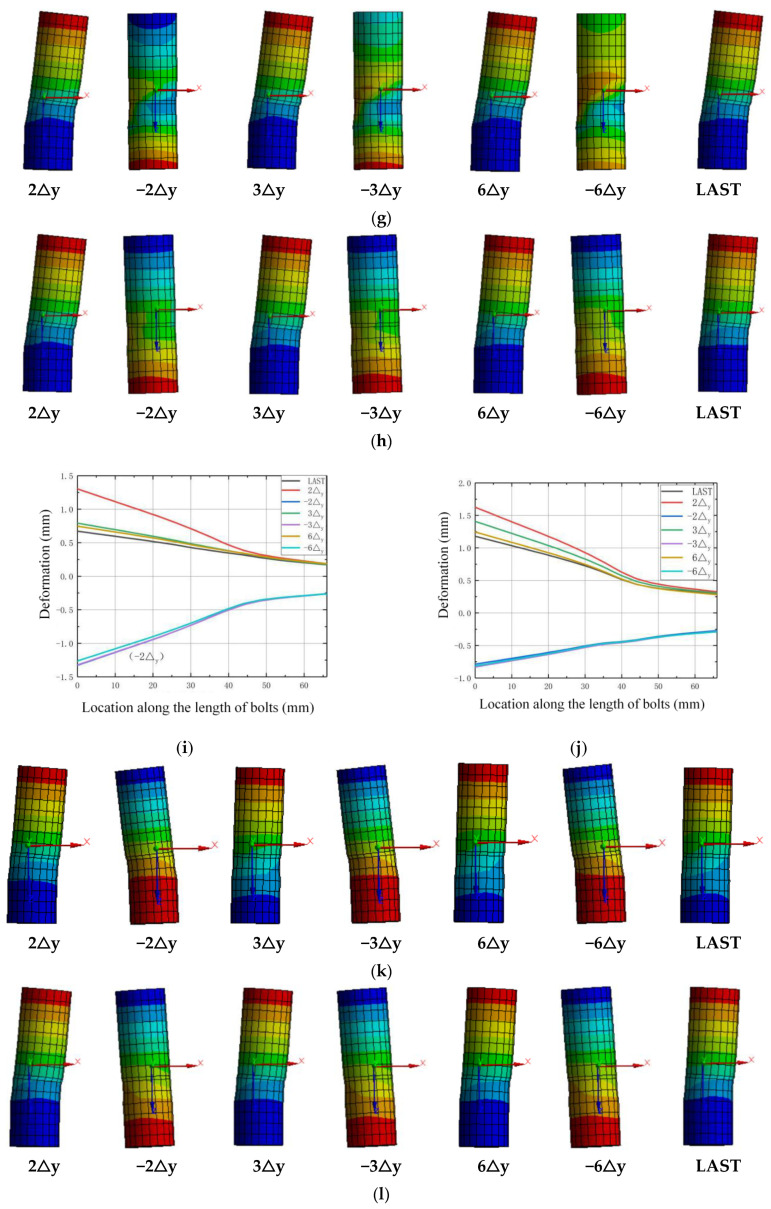

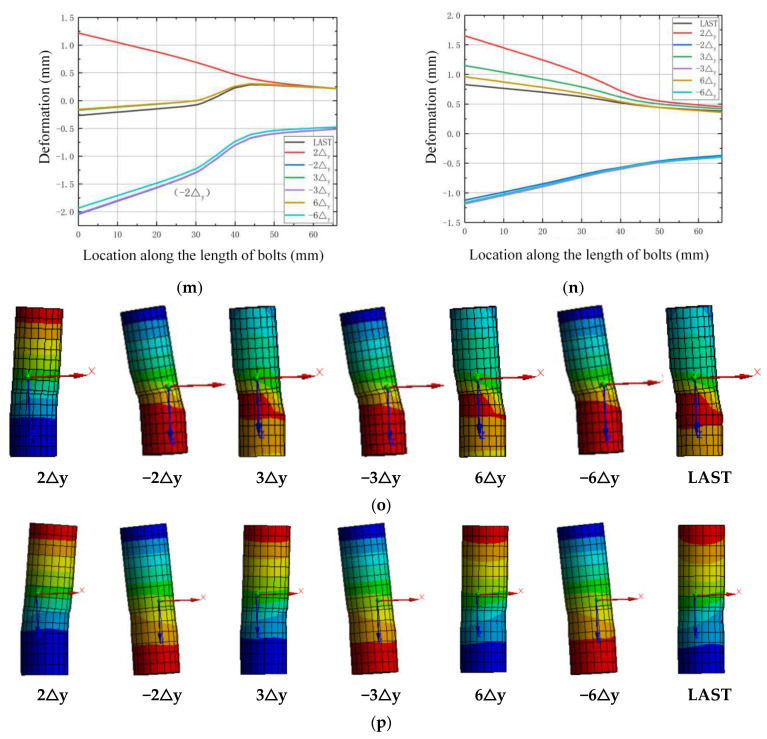
Deformation of the bolts for convex connections: (**a**) *n* = 0, bolts 1; (**b**) *n* = 0.2, bolts 1; (**c**) *n* = 0, development of deformation for bolts 1; (**d**) *n* = 0.2, development of deformation for bolts 1; (**e**) *n* = 0, bolts 2; (**f**) *n* = 0.2, bolts 2; (**g**) *n* = 0, development of deformation for bolts 2; (**h**) *n* = 0.2, development of deformation for bolts 2; (**i**) *n* = 0, bolts 3; (**j**) *n* = 0.2, bolts 3; (**k**) *n* = 0, development of deformation for bolts 3; (**l**) *n* = 0.2, development of deformation for bolts 3; (**m**) *n* = 0, bolts 4; (**n**) *n* = 0.2, bolts 4; (**o**) *n* = 0, development of deformation for bolts 4; (**p**) *n* = 0.2, development of deformation for bolts 4.

**Figure 21:**
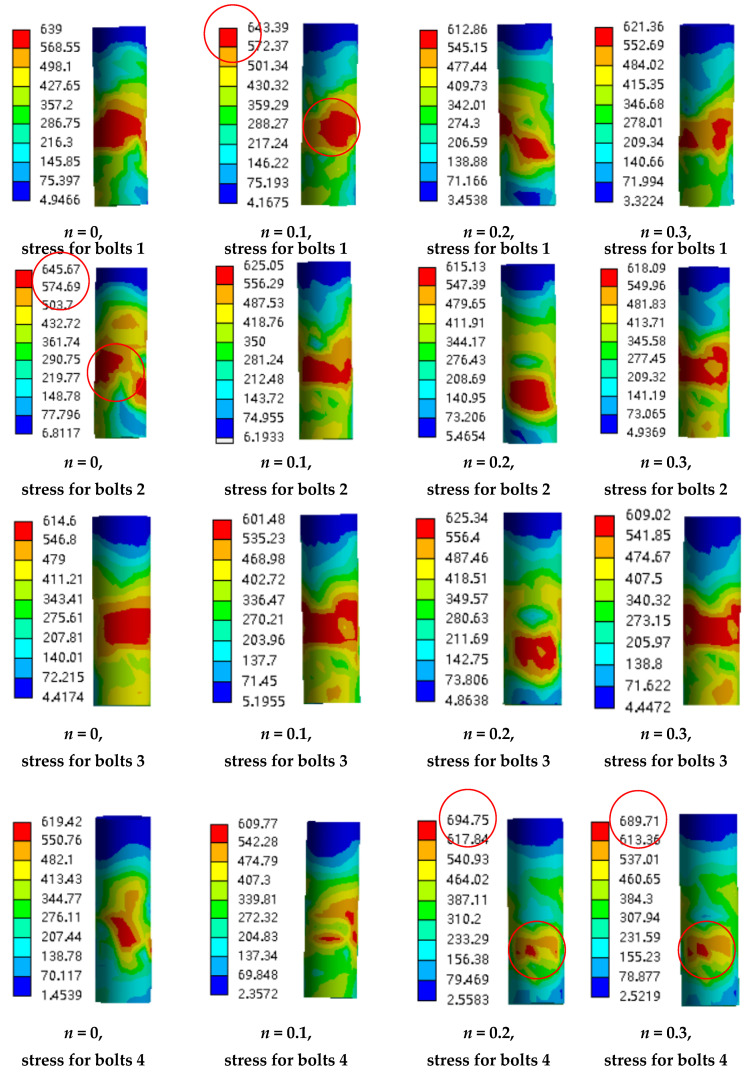
The von Mises stress of the bolts for convex connections.

The deformation and stress state of high-strength bolts during the loading process are investigated to check the working state. The row of bolts are numbered as 1, 2, 3, and 4 along the direction of the horizontal load from the left to the right. Figure 18, Figure 19, Figure 20 and Figure 21 show the deformation along the horizontal direction (x direction) and the von Mises stress of each row of bolts when the loading step reaches 2Δ_y_, −2Δ_y_, 3Δ_y_, −3Δ_y_, 6Δ_y_, −6Δ_y_, and the end of the last load step.

It is shown in Figure 18 and Figure 20 that the deformation of the bolts 1 and bolts 2 are nearly symmetric with bolts 4 and bolts 3, respectively, and the deformation generally decreases as the axial compression ratio increases. It is shown in Figure 19 that the maximum von Mises stress of the bolts for concave connections during the loading process is 625.66 Mpa, which is smaller than the yield stress with 640 MPa, and this means that all the bolts are under elastic state during the loading process. However, for convex connections, it is shown in Figure 21 that the maximum von Mises stress of some of the bolts exceeds the yield stress, which means that some of the bolts are under plastic state during the loading process. Thus, some of the bolts need to be replaced after large earthquakes for convex connections. It can also be observed from Figure 19 and Figure 21 that the axial compression ratio has little effect on the stress development of both kinds of connections.

## 5. Conclusions 

Refined FEA models are built to investigate the seismic behavior of the proposed two kinds of slip-friction connections with different axial compression ratios, and the conclusions can be summarized as follows:

(1) Both kinds of connections show good energy dissipation behavior, and the increase of axial compression force can increase the energy dissipation ability of the connections;
(2) The *M-θ* curves of the convex connection are smoother and steadier than those of the concave connection;
(3) The energy dissipation ability of the proposed connections is better than that of the traditional AFC, especially when the axial compression force is applied to the column;
(4) All the components of the connections except for some of the bolts in the convex connection are under elastic state during the whole loading process, which means that the two kinds of connections can achieve the objective of damage-free energy dissipation.

## 6. Recommendations for Future Research 

It is worth noting that all the conclusions here are based on the FEA results while there are no corresponding tests. The authors have designed the specimens and the tests will be conducted in the laboratory to validate the FEA results in the future.

Furthermore, the research is mainly focused on the seismic behavior of the proposed column base connection while further researches should be conducted to study the effect of the connection on the whole structure, such as the effects of displacements on the bending moment and shear in the rest of the structure.

In addition, external force is needed to push the column with the proposed connections back to the initial vertical state, which is not convenient. Therefore, re-centering systems should be developed for this kind of connection to help the structure back to the initial state after earthquakes.

## Figures and Tables

**Table 1:** Material properties of the steel.

Steel Type	Elastic Modulus *E*/GPa	Yield Strength *f_y_*/MPa	Tensile Strength *f_u_*/MPa	Poisson’s Ratio υ
High-strength bolt	206	640	800	0.3
Other steel	206	345	560	0.3

**Table 2:** Value of the axial vertical load.

*n*	0.1	0.2	0.3
Vertical load (kN)	409	817	1227

**Table 3:** The equivalent viscous damping ratio *h*_e_.

*n*	*h_e_*
0	0.55
0.1	0.63
0.2	0.72
0.3	0.81

**Table 4:** Δ_s1max_ of upper arc endplate for both kinds of connections.

*n*	Δ_s1max_	Δ_s1max_/W
0	0.773	1/647
0.1	0.684	1/731
0.2	0.541	1/924
0.3	0.497	1/1006

## Abbreviations

AFC	asymmetric friction connection
FEA	finite element analysis
SHJ	sliding hinge joint
*B*	width of the flange
*E*	elastic modulus
*f_y_*	yield strength
*f_u_*	tensile strength
*H*	height of the H-shaped section
*h*_e_	equivalent viscous damping ratio
*M*	moment at the rotation center
*n*	axial compression ratio
*R*	inner radius of the arc endplates
*t*_f_	thickness of the flange
*t*_w_	thickness of the web
*W*	width of the arc endplates
υ	Poisson’s ratio
*θ*	rotation angle of the connection
Δ_y_	displacement of the top of the column corresponding to a rotation angle of 0.006 radianΔ_s1max_: maximum deformation of the upper arc endplate
